# Direct energy consumption by inhibitory GABA_*A*_ receptors: from synaptic mechanisms to network activity

**DOI:** 10.3389/fnins.2026.1834375

**Published:** 2026-07-28

**Authors:** Sergey A. Menzikov, Danila M. Zaichenko, Aleksey A. Moskovtsev, Sergey G. Morozov, Aslan A. Kubatiev

**Affiliations:** Institute of General Pathology and Pathophysiology, Moscow, Russia

**Keywords:** ATP consumption, Cl^–^, HCO_3_^–^ATPase, energetic balance, GABA_*A*_ receptors, hypothesis, network activity, plasticity

## Abstract

Despite the brain’s use of adenosine triphosphate (ATP) to perform various intracellular processes, its major energy consumption occurs during neuronal communication. It is estimated that the majority of energy expenditure occurs at excitatory synapses via the activity of energy-dependent pumps (ATPases), which are involved in maintaining and restoring ion gradients after receptor activity. Inhibitory synapses are believed to contribute insignificantly to energy consumption because of their indirect energy consumption via cation-coupled chloride cotransporters (CCCs) and the subsequent function of Na^+^, K^+^ ATPase. Recent findings concerning the properties of inhibitory receptors suggest that they directly contribute to energy consumption. Fast synaptic inhibition in the brain is mediated by type A GABA_*A*_ receptors (GABA_*A*_Rs), which are ligand-gated, anion-selective channels that are open upon the binding of the neurotransmitter and that play a key role in regulating neuronal excitation. Impairment of their function during network/seizure activity is accompanied by changes in the ionic and energy levels in neurons, the mechanisms of which remain largely unclear. Clarification of these processes is crucial for the treatment of neurodegenerative disorders that are linked with dysregulation of the inhibition/excitation (I/E) balance and energy deficiency. Recent evidence has shown the presence of an ATP-hydrolyzing site in the structure of GABA_*A*_Rs, which is involved in network activity. We hypothesized that this ATPase activity can directly contribute to energy use. This hypothesis is supported by data indicating that γ-phosphate analogs targeting distinct ATP binding and hydrolysis stages can reduce the receptor function under certain conditions. Moreover, some GABA_*A*_Rs assemblies have the capacity to participate in both the passive and active transport of Cl^–^ against the electrochemical gradient. Finally, under conditions of brain hypoxia or glucose deficiency syndrome, the function of inhibitory neurons is primarily impaired. Here, we explore the ATPase hypothesis that some GABA_*A*_R subtypes may be directly involved in reducing the energy budget, and we present arguments for and against this theory while highlighting gaps in our knowledge of this neurobiological process.

## 1 Introduction

**FIGURE 1:**
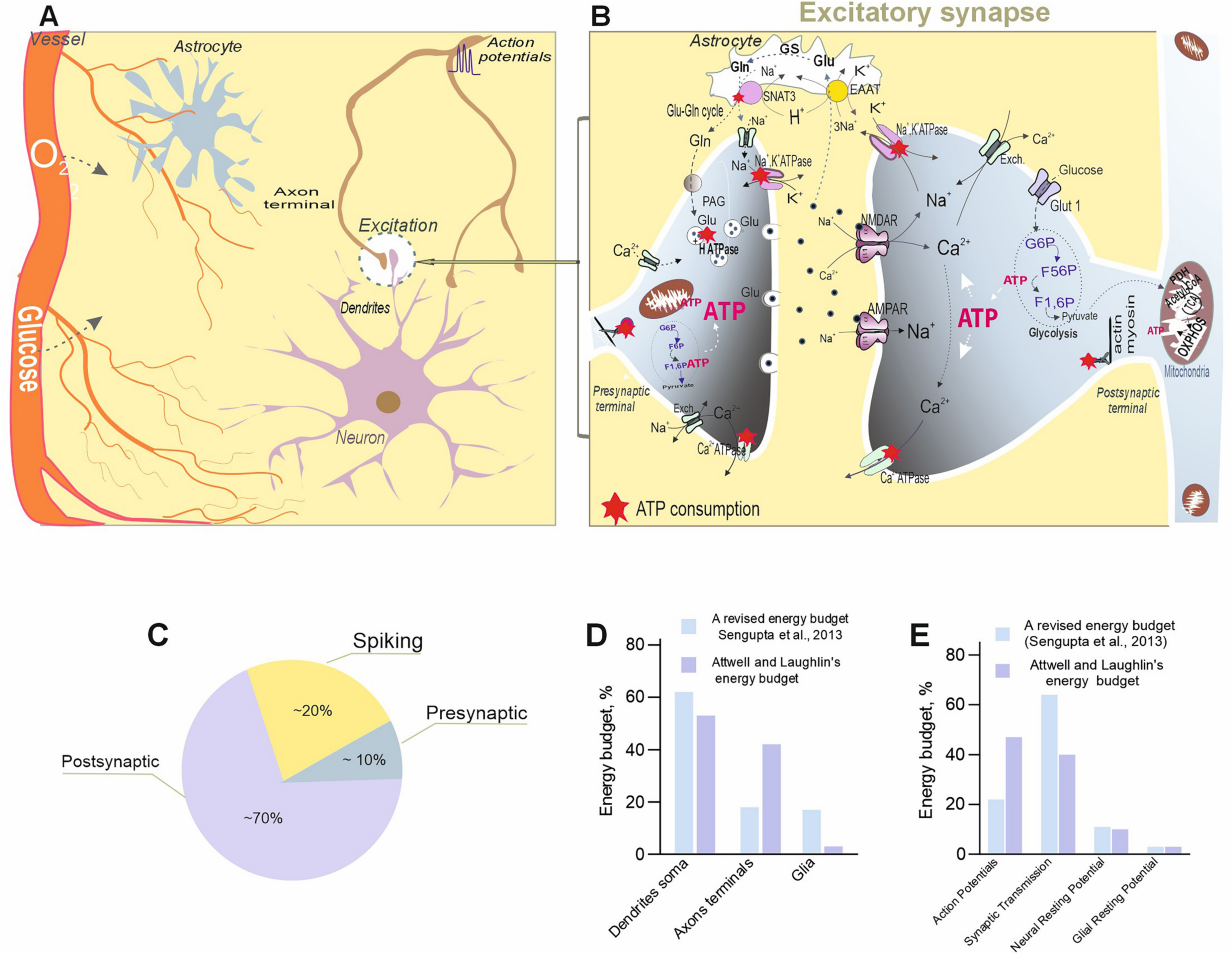
Energy use by excitatory neurons. **(A)** Energy is delivered to the brain as oxygen (O_2_) and glucose from blood vessels. This figure shows a microcircuit in which an axonal input activates by action potentials an excitatory synapse onto an output cell. **(B)** Figure shows pre- and postsynaptic terminals of excitatory synapse as well as astrocyte. The release of glutamate (Glu) from synaptic vesicles into the synaptic cleft evokes a brief change in the concentration of Glu, which diffuses out of the cleft in *msec* to surrounding regions and activates low-affinity AMPA receptors and high-affinity NMDA receptors of the postsynaptic terminal. The astrocyte Glu transporters (EAAT1 and EAAT2) maintain the concentration at a low level. The Glu is then converted into glutamine (Gln) by glutamine synthetase (GS). Astrocytes excrete Gln back into the extracellular media through the Na^+^ driven SNAT3 transporter, which is taken up bu an as yet unconfirmed neuronal Gln transporter of the presynaptic terminal. Neurons convert Gln to Glu via a phosphate-activated glutaminase (PAG) reaction to replenish their vesicular Glu stores. Glucose is transported into neurons primarily by the Glut 3 transporter. ATP is supplied to presynaptic and postsynaptic terminals via glycolysis in the cytosol and oxidative phosphorylation in mitochondria. Structures that consume energy at excitatory synapses include (i) presynaptically after Ca^2+^ entry via voltage-gated Ca^2+^-channels to trigger vesicle release, pumping of neurotransmitter into vesicles, and on the vesicle cycle and H^+^ATPase involvement; (ii) pumping Na^+^ and K^+^ by Na^+^, K^+^ATPase, and Ca^2+^ by Ca^2+^ATPase to restore ion gradients postsynaptically after ion entry via AMPA and NMDA receptors and Ca^2+^ release from internal stores; (iii) in axons after action potentials (APs); (iv) in astrocytes to power the glutamate–glutamine cycle; and (v) in actin/myosin function–ATP is bound and hydrolyzed by the myosin head, breaking the bond between myosin and actin. The energy released cocks the myosin head into a “high-energy” position, ready to bind to actin again (Harris et al., 2012). **(C)** Relative energy expenditure in presynaptic and postsynaptic terminals as well as during spiking (Attwell and Laughlin, 2001; Harris et al., 2012). **(D)** Comparative analysis of energy consumption in dendrites, axons and glia in the original version (Attwell and Laughlin, 2001) and in the revised version (Sengupta et al., 2013). **(E)** Comparative analysis of ATP expenditure by action potentials, synaptic transmission, neuronal and glial resting potential.

**FIGURE 2:**
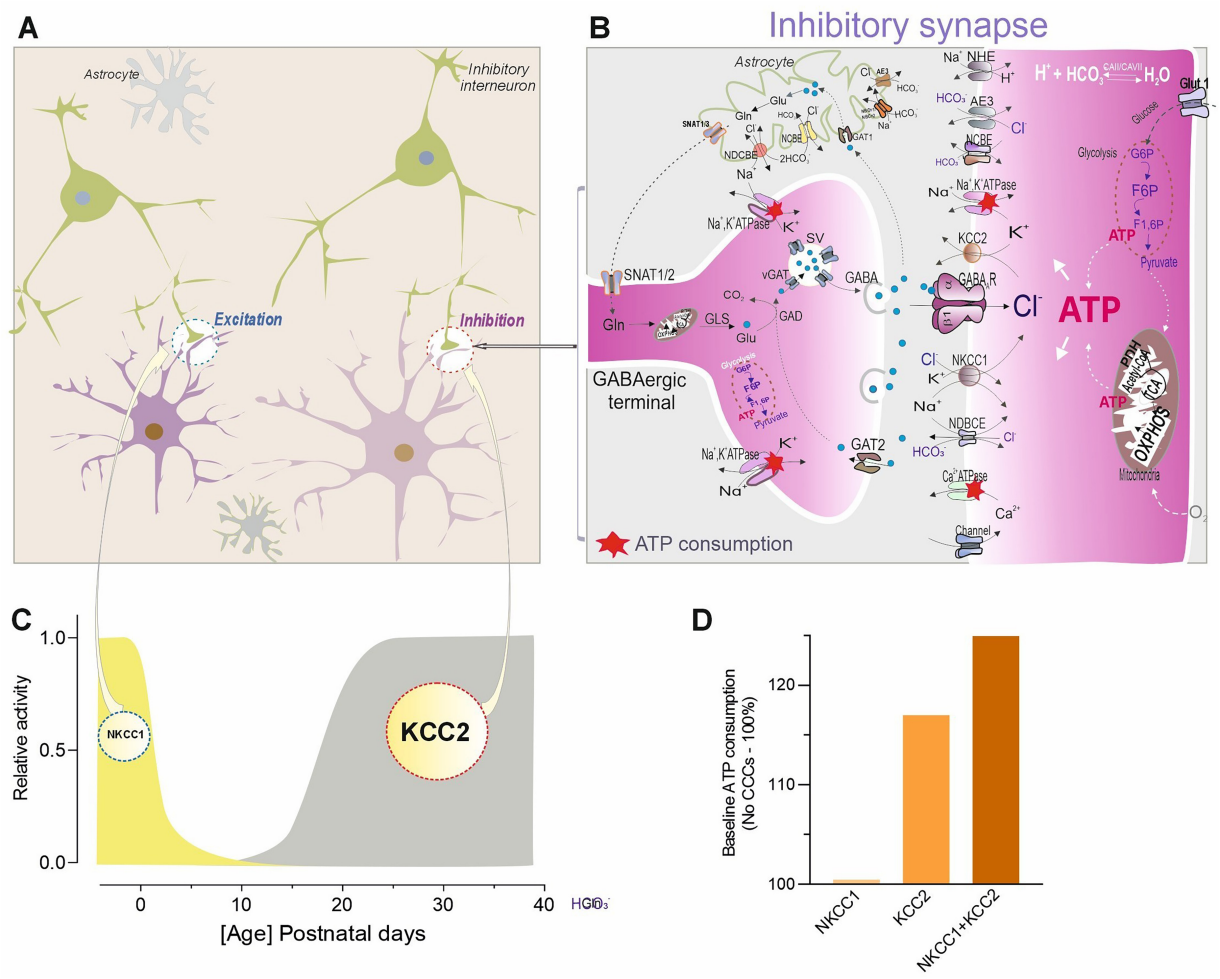
Structures that consume ATP by inhibitory signaling. **(A)** This figure shows a microcircuit in which inhibitory neuron’s axon fires and releases neurotransmitters at a synapse. Depending on the target cell, the GABA_A_ receptors can mediate inhibition or excitation depending on the [Cl^–^]_i_. **(B)** Glutamine (Gln) is released by the astrocyte via SNAT3 transporter and is further imported by SNAT1/2 into the GABAergic neuron, where it is metabolized to glutamic acid (Glu) by glutaminases GLS1/2. GAD67/65 converts Glu into GABA (blue circles), which is then loaded into the synaptic vesicles (SV) by VGAT, trafficked to the presynaptic membrane, and released into the synaptic cleft. Once released, GABA interacts with GABA_*A*_Rs, activating Cl^–^ influx into neuron. The excess of GABA is taken up by the astrocyte via GAT1/3, which simultaneously translocates 2 Na^+^ and one Cl^–^. In the astrocyte, GABA is sequentially catabolized by GABA transaminase ABAT and SSA dehydrogenase SSADH into succinic acid, which further enters the Krebs cycle, where it is processed into α-ketoglutarate, the precursor of Glu and Gln. In inhibitory synapses the ATP is used on: (i) cation pumping by Na^+^, K^+^ATPase to restore ion gradients postsynaptically after Cl^–^ ion entry via GABA_A_ receptors and recovery of [Cl^–^]_i_ by K^+^/Cl^–^ cotransporter (KCC2) and Na^+^/K^+^/Cl^–^ cotransporter (NKCC1); (ii) presynaptically after Ca^2+^ entry to trigger vesicle release, pumping of neurotransmitter into vesicles, and also on the vesicle cycle; (iii) in axons after action potentials (APs); (iv) in astrocytes to power the glutamate–glutamine cycle. Membrane transporters–Na^+^-driven Cl^–^/HCO_3_^–^ exchanger (NDBCE); Na^+^-dependent HCO_3_^–^ transporter (NCBE); Anion exchange protein (AE3); Electrogenic Na^+^/H^+^ exchanger (NHE). Buffering by CO_2_/HCO_3_^–^ is supported via the action of enzymes from the CAs family. **(C)** Schematic representation of the role of NKCC1 and KCC2 function from the cerebral cortex during the embryonic and postembryonic development, and their role in the receptor excitation or inhibition, respectively. **(D)** Calculation of ATP consumption by CCCs activity (Attwell and Laughlin, 2001).

Adenosine triphosphate (ATP) is the main energy substance that ensures physiological and biochemical processes in cells of various natures (Du et al., 2008; Kobayashi et al., 2013). Although neurons use energy for protein and lipid synthesis, a significant portion of the energy budget is consumed by ensuring the trafficking of subcellular organelles and the homeostatic mechanisms of network activity (Gustafsson et al., 2024; Pepperell, 2024; Qi et al., 2020). Preliminary calculations on the distribution of ATP consumption across various structures and mechanisms that contribute to synaptic transmission have revealed that the restoration of ion homeostasis by transport ATPases and the generation of postsynaptic responses consumes most of the energy at excitatory synapses (Figure 1; Attwell and Laughlin, 2001; Wang et al., 2017). It has been hypothesized that, in contrast to excitatory synapses, inhibitory synapses do not significantly consume energy because of the proximity of chloride ion (Cl^–^) reversal to the resting membrane potential (Harris et al., 2012). In particular, the calculations demonstrated that inhibitory synapses use energy indirectly, namely, via the activity of secondary-active chloride transport systems (CCCs) and subsequently via Na^+^, K^+^ ATPase activity, thereby contributing to the recovery of ion homeostasis in neurons (Figure 2; Schmidt et al., 2018; Schulte et al., 2018). However, in these research disregards the fact that the driving force (DF_*Cl*_^–^) for Cl^–^ is augmented by excitatory input and that the resulting “shunting” inhibition engenders Cl^–^ inflow (Doyon et al., 2016). Furthermore, evidence has shown that network activity is accompanied not only by an increase in the intracellular chloride concentration ([Cl^–^]_*i*_) but also by a decrease in the neuronal pH (pH_*i*_) and a reduction in the ATP concentration ([ATP]_*i*_) (Shetty et al., 2012; Halverson et al., 2023).

The GABAergic system is a complexly organized network of various neurons. The interaction of a mediator with GABA_*A*_ receptors (GABA_*A*_Rs) facilitates the rapid influx of Cl^–^ into cells along an electrochemical gradient (Ghit et al., 2021). This influx in turn results in the inhibition of membrane potential (*V*_*m*_) (Figure 2A). As evidenced by preliminary studies of receptor properties, their function is supported not only through phosphorylation processes by protein kinases (PKs) (Nakamura et al., 2015) but also through ATPase activity (Table 1; Stelzer et al., 1988). The PKs comprise a shared catalytic domain that facilitates the transfer of γ-ATP to a serine (Ser), threonine (Thr), or tyrosine (Tyr) residue of the designated protein. In contrast, ATPase is capable of forming a high-energy acyl phosphate bond through ATP hydrolysis (Kühlbrandt, 2004; Menzikov and Morozov, 2019). The involvement of ATP in maintaining inhibitory signaling is supported by data indicating that γ-phosphate analogs targeting distinct ATP binding and hydrolysis stages can reduce receptor function during their massive activation (Chase et al., 2018). Several studies have demonstrated the existence of receptor subtypes that, as suggested, can be involved in Cl^–^ transport against the electrochemical gradient via phosphorylation processes (Rapallino et al., 1999). Moreover, hypoxia and Glucose Transporter 1 (GLUT1)-Deficiency Syndrome (GLUT1-DS) predominantly affect inhibitory neurons, thereby substantiating their pivotal function in energy expenditure (Laschet et al., 2007; Rajasekaran et al., 2022; Schoknecht et al., 2025). Thus, the accumulated data concerning the properties of inhibitory synapses allow us to hypothesize that they directly and significantly contribute to energy consumption.

**TABLE 1:** Involvement of ATP in the rundown of GABA_A_ receptor’s function *in vitro* and pathological conditions.

Preparation/model system	Condition causing rundown or ATP dependence	Key finding	References
In vitro			
GABA_*A*_R function in hippocampal cells	The absence of intracellular ATP	Phosphorylation by ATP hydrolysis	Stelzer et al., 1988
Dissociated nucleus tractus solitarii neurones	The absence of intracellular ATP	Intracellular ATP (2 mM) and Mg^2+^ prevent rundown	Shirasaki et al., 1992
Chick cortical neurons	Translational or post-translational mechanisms	Agonist-dependent down-regulation	Baumgartner et al., 1994
GABA_*A*_R responses in isolated bullfrog dorsal root ganglion neurons	The absence of intracellular ATP	Receptor function is modulated by cGMP	Bradshaw and Simmons, 1995
Recombinant GABA_*A*_Rs αlβ2y2 and α6β2y2	Ultra-rapid application with a piezo electric translator of 1 mM GABA to mammalian cells	Distinct deactivation and desensitization kinetics	Tia et al., 1996
Rat cerebellar granule cells	The absence of intracellular ATP	Tyrosine phosphorylation is controlled by PTPs	Amico et al., 1998
Rat hippocampal neurons	Repetitive stimulation with 1 μM muscimol	Destruction of microtubules, as or actin fibers, caused a rundown	Meyer et al., 2000
GABA_A_ current in α1β2	GABA current rundown	Phosphatases inhibitors can prevent the current rundown	Cifelli et al., 2021
Disorders			
Acutely dissociated CAl pyramidal neurons of the rat	Ischemia induces the rundown	Rundown is triggered by a decrease in [ATP]_i_	Harata et al., 1997
Mammalian CNS neurons during experimental anoxia	Decreasing intracellular ATP synthesis	Rundown was prevented by adding ouabain or SPAI-I (Na^+^, K^+^ ATPase inhibitor-I)	Akaike, 1995
Neurons in the nucleus of the solitary tract (NTS)	Renal-wrap hypertensive rats	Repetitive application of GABA revealed a decline	Tolstykh et al., 2003
α1-subunit GABA_*A*_R	Human partial epilepsy	Maintaining function by phosphorylation	Laschet et al., 2007
GABA_*A*_R-microtransplanted to *Xenopus* oocytes	Repetitive activation of receptor temporal lobe epilepsy	Rundown was significantly enhanced by Zn^2+^ and abolished by gabazine	Palma et al., 2007
GABA_*A*_Rs-microtransplanted to *Xenopus* oocytes	Human epileptic brains	Adenosine receptor blockers alter the stability of receptors	Roseti et al., 2008
Hippocampus neurons	Repetitive activation of GABA_*A*_Rs temporal lobe epilepsy	Enhanced rundown at the first spontaneous seizure	Mazzuferi et al., 2010
Human hypothalamic hamartomas/expression in *Xenopus* oocytes	Treatment-resistant gelastic seizures	Rundown was attenuated by ATP or okadaic acid	Li et al., 2011
Cerebellar glial cells β3y2 > α2β3y2 > α2β1y2	Via promiscuity with the vasoactive peptide Urotensin II receptor	Rundown involves Ca^2+^ and phosphorylation	Desrues et al., 2012
Hippocampal GABA_*A*_Rs (temporal lobe epilepsy)	Repetitive activation of GABA_*A*_R temporal lobe epilepsy	Changes in the sensitivity of rundown to drug treatments	Cifelli et al., 2013
Suprachiasmatic nucleus	Sustained GABA_*A*_R activation	A novel mechanism was identified	Hummer et al., 2015
GABA_*A*_Rs-microtransplanted to *Xenopus* oocytes	Human drug-resistant mesial temporal lobe epilepsy	Rundown is associated with human lobe epilepsy	Ragozzino et al., 2005
Neuronal or recombinant GABA_*A*_Rs	Human epileptic brain or plasma membrane of *Xenopus* oocytes	Vanadate and okadaic acid inhibited rundown	Palma et al., 2004

The molecular and circuit mechanisms underlying ATP use during GABAergic transmission are challenging to investigate because of the diversity of properties of receptor subunits and their dual functionality. Specifically, in contrast to inhibition, GABA_*A*_Rs can mediate the depolarization of V_*m*_ not only during early neuronal development but also in adult brains during massive activation by agonists (Menzikov et al., 2025). This depolarization, which can turn into neuronal excitation, is of particular interest because it is coupled with increased energy consumption and, as a result, with the manifestation of a number of neurodegenerative disorders (such as seizures) (Fujiwara-Tsukamoto et al., 2003; Sørensen et al., 2018; Arosio and Musio, 2025). Currently, there are two hypothetical models for the ionic basis of GABA_*A*_-mediated depolarization. Notably, these two models are not absolutely mutually exclusive since they suggest a collapse of the Cl^–^ gradient and simultaneous outflow of bicarbonate ions (HCO_3_^–^) from neurons. However, a closer inspection of these models reveals notable discrepancies. In one model, Cl^–^ enters neurons via the Cl^–^ receptor pore during depolarization (Staley et al., 1995), whereas in another, Cl^–^ is released from cells (Perkins, 1999). These observations suggest the presence of divergent pathways for restoring [Cl^–^]_*i*_. In summary, a minimal consensus version of both theories posits that during GABAergic depolarization, anion gradients collapse. However, the correction of this collapse will require the functioning of various transport systems for rapid restoration of intracellular anion homeostasis after receptor activity. Specifically, in the Cl^–^-accumulating model, the primary role in restoring ion homeostasis is assigned to CCCs (Chamma et al., 2012), whereas in the alternative model, the presence of ATPase was considered (Perkins and Wong, 1996). However, despite the dominant view of the primary role of CCCs in the restoration of [Cl^–^]_*i*_, there is a debate in the literature regarding the inconsistencies between their transport kinetics and the rate of restoration for receptor activity and ion homeostasis (Doyon et al., 2016; Lombardi et al., 2019). The contradictions between the functions of CCCs in short- and long-term ionic plasticity, in conjunction with the substantial body of evidence pertaining to the role of ATP and ATPase in preserving receptor functionality, have prompted an analysis into the role of inhibitory synapses in direct and substantial energy expenditure.

In this review, the findings related to the role of energy consumption during inhibitory synaptic transmission were summarized. The initial presentation is of the fundamental processes that ensure the formation of the energy balance in cells and systems involved in energy consumption in excitatory and inhibitory synapses. Afterward, we present arguments for and against where and under what circumstances GABA_*A*_Rs are involved in direct ATP consumption. Lastly, we examine the role of GABA_*A*_R_*ATP*_ subtypes in energy expenditure during seizure activity.

## 2 Energy budget

Neuronal ATP concentrations ([ATP]_*i*_) and/or phosphorus inorganic (P_*i*_) can be regarded as the cellular energy status (or cellular energy balance) reflecting both energy supply mainly via glycolysis and oxidative phosphorylation (Figures 1B, 2B; Yates, 2024; MacVicar et al., 2017; Lerchundi et al., 2020). To ensure high energy demands, the brain uses mainly glucose (Yates, 2024). Glucose is a monosaccharide whose catabolism in the cells during glycolysis results in the formation of pyruvate and ATP. However, a majority of ATP in the brain is formed in the mitochondria through oxidative phosphorylation of ADP–a process that occurs in the mitochondrial electron transfer chain (Figure 1B; Casanova et al., 2023; Duarte et al., 2023). Oxidative phosphorylation is able to provide high ATP yields, whereas glycolysis is a fast ATP production pathway with a smaller increase. Taken together, the [ATP]_*i*_ levels (approximately, 2–4 mM) could be regarded as the cellular pool of energy (Erecińska and Silver, 1989; Rangaraju et al., 2014; Imamura et al., 2009; Kitajima et al., 2020). In the CNS, various ATP-generating systems and processes performance “on demand,” depending on the local levels of synaptic activity. As example, basal [ATP]_*i*_ production can increase (∼15%) with increasing activation (Shetty et al., 2012; Bergmeister et al., 2025). Oxidative phosphorylation is able to provide high ATP yields, whereas glycolysis is a fast ATP production pathway with a lower ATP production net gain (Bergmeister et al., 2025). It has been widely acknowledged that energy deficits are associated with neurodegenerative disorders. These energy deficits might result from impairments in glycolysis and/or mitochondrial ATP production pathways.

### 2.1 Glycolysis

Glycolysis occurs in the cytosol and which is a chain of reactions carried out mainly by ten enzymes that results in the formation of two pyruvate molecules and two ATP molecules. Pyruvate molecules also can be imported into mitochondria, where they are converted into acetyl-CoA, which is integrated into the tricarboxylic acid (TCA) cycle, from which two ATP molecules are produced (per glucose molecule). Glucose oxidation starts with its phosphorylation into glucose-6-phosphate by hexokinase. Under anaerobic conditions, pyruvate can then be converted into lactate through lactate dehydrogenase complex. Glycolysis becoming the predominant process of ATP generation only when neurons become active and have increased energy needs (Cunnane et al., 2020), which use glucose and lactate to drive glycolysis and the TCA cycle (Figure 1B; Faria-Pereira and Morais, 2022). While, recent discoveries suggested a more complex picture of ATP generation in neurons - aerobic glycolysis is the principal means of generating ATP in neuronal cell bodies under basal conditions and in activated states (Yates, 2024). Impairments in glycolysis directly compromise GABA_*A*_Rs function, as receptor phosphorylation and stability depend on ATP produced by membrane-associated glycolysis enzymes like GAPDH (glyceraldehyde-3-phosphate dehydrogenase) (Laschet et al., 2007; Hartman, 2017). Moreover, in epilepsy, impaired glycolysis (specifically reduced ATP production) leads to dysfunction of GABA_*A*_R, reducing inhibitory tone and increasing excitability. Furthermore, hypoxia, as well as GLUT1-DS, primarily affects inhibitory neurons (Klepper et al., 2020; Kloiber et al., 1993). Specifically, in a mouse model of Glut 1, the authors showed no energy metabolism impairments, whereas synaptic inhibition of cortical pyramidal and thalamic cells was reduced, confirming the important role of energy substrates in maintaining their functional activity (Rajasekaran et al., 2022).

### 2.2 Oxidative phosphorylation

A majority of ATP in the brain is formed in the mitochondria through oxidative phosphorylation (OP) of ADP − a process that occurs in the mitochondrial electron transfer chain (Figures 1B, 2B; Casanova et al., 2023; Duarte et al., 2023). The pyruvate molecules formed in glycolysis can be imported to the mitochondria and generate acetyl-CoA molecules, which will enter the TCA cycle. From this cycle, ATP, but mainly NADH and FADH_2_, electron carriers are generated and used to fulfill the OP complexes chain, where, ultimately, Complex V (ATP synthase) generates ATP from ADP and inorganic phosphate. Glycolysis is a core energy-producing pathway in cells; it converts glucose to two net ATPs and pyruvates, which can then be utilized by the mitochondria to generate an additional 34 ATPs through oxidative phosphorylation in the TCA cycle (Mookerjee et al., 2017). From this cycle, the electron carriers NADH and FADH_2_ are synthesized and can deliver electrons to the electron transport chain Complex I and II, respectively. When electrons arrive at Complex IV, electrons and protons are conjugated with O_2_, originating H_2_O. Simultaneous to electron transfer between electron transfer chain complexes, protons accumulate in the mitochondrial intermembrane space and are used by Complex V (ATP synthase complex) to produce about twenty eight ATP molecules per glucose molecule, in a pathway designated OP (Figure 1). Thus, the last enzyme of the electron transfer chain, and therefore the ulterior generator of ATP, is the ATP synthase (F_1_F_0_-ATPase) (Patro et al., 2021). Mitochondria within synaptic structures are key for maintenance of functional neurotransmission and this process is modulated by energy metabolism, mitochondrial distribution, mitochondrial trafficking, and cellular synaptic calcium flux (Faria-Pereira and Morais, 2022). Mitochondrial dysfunction is a key driver of a number of pathologies due to the brain’s high energy demands and reliance on mitochondrial homeostasis (Ren et al., 2024; Cardanho-Ramos and Morais, 2021). Other authors suggested the crucial role of GABAergic neurons in detecting mitochondrial stress and orchestrating non-cell-autonomous changes throughout the organism (Rathor et al., 2024). Kanellopoulos and colleagues discovered that mitochondrial hyperactivity in GABAergic neurons contributes to social behavioral impairments by redistributing neurotransmitter from synaptic compartments to mitochondria via Aralar carrier (Kanellopoulos et al., 2020).

## 3 Synaptic transmission and energy use

The brain is dependent on a significant amount of energy to function. However, the brain energy expenditure is quite heterogeneous: it varies between different brain regions, such as in white vs. gray matter (Attwell and Laughlin, 2001; Harris et al., 2012); between types of cells, and between type of neurons (excitatory or inhibitory) (Yuan et al., 2018; Qi et al., 2020). Neurons have morphologically and functionally distinct subcompartments, differentiating degree of energy expenditure: dendrites soma (∼50%–60%), axons (∼18%–40%) and presynaptic terminals (Figure 1C; Faria-Pereira and Morais, 2022). When estimating the brain’s energy budget, synaptic transmission processes, which include postsynaptic currents, neurotransmitter recycling and presynaptic Ca^2+^ fluxes, are considered as the main energy-consuming process.

### 3.1 Excitatory synapses

Most of the brain’s energy (80%) is spent in synapses, with most of them (about 85%) being excitatory synapses (Sengupta et al., 2013; Harris et al., 2012; Lennie, 2003; Lange et al., 2015; Figures 1C, D). Calculations predict that the pre- and postsynaptic exciting mechanisms mediating synaptic transmission (including glutamate accumulation in vesicles) consume more than half of the total ATP (Figure 1C). Specifically, from neuronal activity, the largest energy is expended by action potentials and postsynaptic potentials of total signaling-related usage, much less are neuronal and glial resting potentials, presynaptic calcium entry and neurotransmitter recycling are third, and least demanding are calcium transients in spines and vesicle recycling (Figure 1E; Attwell and Laughlin, 2001; Harris et al., 2012; Pathak et al., 2015). However, maintaining ion homeostasis in neurons and the recovery of cations level that generate postsynaptic responses during of the receptor activity by membrane ion pumps (transport ATPases) requires the greatest expenditure of energy (Attwell and Laughlin, 2001; Figure 1E). The vesicular H^+^ATPase, which is responsible for the acidification of presynaptic vesicles that drives the uptake of neurotransmitters like glutamate. The Ca^2+^ATPase restores Ca^2+^ after intracellular transients (Kühlbrandt, 2004; Figure 1B). While, Na^+^, K^+^ATPase restores the transmembrane Na^+^/K^+^ gradients that were changes by neuronal firing due to action potentials, and to sustain the resting potential (22% and 20% of energy consumption, respectively) (de Lores Arnaiz and Ordieres, 2014). The Na^+^, K^+^ATPase consumes exactly 1 ATP molecule per cycle. During each cycle, the enzyme hydrolyzes the ATP to actively transport three Na^+^ out of the cell and two K^+^ into the cell against their concentration gradients (Meyer et al., 2022). At the cellular and whole-body levels, this continuous maintenance of ion gradients and cell volume translates to a massive energy burden (Bo et al., 2023). The Na^+^, K^+^ ATPase/Ca^2+^ATPase are among the largest energy consumers and together can consume up to ∼70%. They typically consume 20%–40% of a cell’s resting energy budget. Meanwhile, at the post-synaptic neuron, ATP is primarily used to extrude ions participating in post-synaptic currents–about 50% of the energy budget consumption (Harris et al., 2012; Howarth et al., 2012). It was found that to match neural energy levels, only a part of the synapses received presynaptic signals during a given period so that neurons have a low action potential frequency (Figure 1E). That is, the number of simultaneously active synapses over a period of time should be adapted to neural energy levels.

#### 3.1.1 Presynaptic vesicle cycling

Presynaptic vesicle cycling is the fast, highly regulated process of neuronal vesicles releasing neurotransmitters via fusion/exocytosis (release of neurotansmitters) and immediately recycling via endocytosis (vesicle retrieval) to maintain synaptic transmission (Rizzoli, 2014; Jiang et al., 2025). Resting synaptic vesicle pools (readily releasable, recycling, reserve) represent a major source of basal energy consumption, adapting to high activity levels by increasing vesicle reuse to manage demands. Proton pump (H^+^-ATPase) uses ATP to create a pH gradient for loading neurotransmitters (Figure 1B; Eaton et al., 2021). Synaptic terminals rely on local glycolysis and mitochondria to meet these high demands, particularly during sustained activity. Mitochondrially derived ATP is rapidly dispersed throughout axons, enabling vesicles to cycle even in boutons lacking mitochondria. Thus the synaptic energy not only drives synaptic vesicle cycle, but also participates in the regulation of this cycle (Mikulasch et al., 2025). Release probability of mediators from presynaptic terminals adapts to synaptic energy levels by regulating the speed of the synaptic vesicle cycle.

#### 3.1.2 Astrocytic

The network activity to elicit local increases in blood flow, glucose uptake, and oxygen consumption are formed not only by neurons but and in the astrocytes, which have endfeet processes close to capillaries (Escartin and Rouach, 2013; Figures 1A, B). Astrocytes are present in higher numbers in species and brain regions that have larger neurons. For example, oligodendrocytes and Schwann cells speed action potential conduction, while astrocytes regulate the ion composition of the extracellular space, take up released neurotransmitters to terminate synaptic transmission, and regulate the development of synapses on neurons. Astrocytes, in the contrast to neurons, consume less ATP (Harris et al., 2012) but contain large intracellular reservoirs of glycogen, which can be broken down to generate glucose-1-phosphate to be fed into glycolysis (DiNuzzo et al., 2010; Lerchundi et al., 2020). An increased glycolytic rate on astrocytes leads to lactate accumulation that is shuttled into neurons for subsequent generation of ATP by oxidative phosphorylation (Magistretti and Allaman, 2018; Hall et al., 2012).

#### 3.1.3 Compartmentalized glycolysis

Usually, under normal conditions, glycolysis enzymes are diffusely localized throughout the cytoplasm (Jang et al., 2016). Hypoxia inhibits the tricarboxylic acid cycle and leaves glycolysis as the primary metabolic pathway responsible for converting glucose into usable energy. However, during hypoxic stress, these enzymes become compartmentalized into cytoplasmic structures since hypoxia inhibits the TCA and leaves glycolysis as the primary metabolic pathway (Fuller and Kim, 2021). Specifically, several studies have revealed that glycolysis enzymes colocalize into glycolytic bodies (G bodies) or analogous condensates in neurons (Jin M. et al., 2017). Such the association of multiple glycolysis enzymes via phase separation may function to enhance the activity of the entire pathway and increase reaction rates critical for ATP production (Yang et al., 2024). Failure to form these condensates results in reduced ATP production and as result, impaired cell viability or impaired synaptic function, demonstrating that compartmentalization is a direct regulatory mechanism of cellular energy homeostasis or impaired synaptic function.

## 4 Inhibitory synapses

If the mechanism of signaling is coupled primarily to a change in postsynaptic receptor conductance or abundance in excitatory synapses; in inhibitory synapses, the mechanism of action due to ionic plasticity can result from postsynaptic changes in the electromotive force for Cl^–^ (DF_*Cl*_−) (Hudson and Grau, 2022). The flux of the hydrophilic Cl^–^ across the hydrophobic plasma membrane occurs exclusively via integral membrane proteins (including, inhibitory receptors) that mediate the transport of Cl^–^ and to a lesser extent HCO_3_^–^ ions (Ghit et al., 2021). The passive Cl^–^ fluxes through anion channels follow DF_*Cl*_−, which depends on the difference between the Cl^–^ equilibrium potential (E_*Cl*_^–^) and the V_*m*_. Therefore, for hyperpolarizing or depolarizing GABA_*A*_R responses necessary that the intracellular Cl^–^ concentration is not in an equilibrium state.

### 4.1 Ionic plasticity

Inhibitory ionic plasticity is based on both short- and long-term shifts in Cl^–^ levels in postsynaptic neurons (Yelhekar et al., 2017; Hudson and Grau, 2022). Active transmembrane structures are required for accumulation or depletion of Cl^–^ from cells (Hübner and Holthoff, 2013). Either primary active Cl^–^ transport, via an ATP-hydrolyzing Cl^–^ pump, or secondary active transport, coupling Cl^–^ transport to the transport of another ions along their gradient, is required to obtain [Cl^–^]_*i*_ below or above this passive distribution. Short-term modulatory changes occur solely to activity-dependent ionic shifts in the neurons (Raimondo et al., 2017), whereas long-term ionic shifts involve changes in kinetic properties or ion-regulatory protein synthesis and trafficking (Kim et al., 2019). In the absence of such active transport processes [Cl^–^]_*i*_ follows a passive distribution, which due to the negative *V*_*m*_ is set at low millimolar concentrations under physiological conditions (given by the Nernst equation). In the presence of active Cl^–^ transport, which is provided by active membrane structures, *V*_*m*_ ≠ *E*_*Cl*_, the difference between these potentials represents the *DF*_*Cl*_^–^ (*DF_*Cl*_^–^* = *V*_*m*_ − *E_*Cl*_^–^*). Minor alterations in the *DF*_*GABA*_ of fundamental GABA_*A*_R-mediated Cl^–^ currents can precipitate substantial modifications in the potency of GABAergic signaling (Rahmati et al., 2018; Watanabe and Fukuda, 2015; Succol et al., 2012; Abruzzo et al., 2021). For instance, if the reversal potential for GABA (*E*_*GABA*_) is more negative than the resting membrane potential, then GABA_*A*_R activation will result in membrane hyperpolarization of *V*_*m*_. However, if the *E*_*GABA*_ is more positive than the resting *E*_*M*_, the stimulation of GABA_*A*_Rs results in a combination of membrane depolarization and shunt inhibition or only excitation if the *E*_*GABA*_ is positive enough (Figures 2A, C).

### 4.2 CCCs and energy consumption

Although various ion channels and transporters are involved in maintaining [Cl^–^]_*i*_ (Rahmati et al., 2018), CCCs play a key role because they generate an electrochemical gradient (Figures 2A–C). In neurons, the major proteins that mediate secondary active transmembrane Cl^–^ transport are Na^+^-dependent K^+^/Cl^–^-cotransporters (NKCC1), K^+^/Cl^–^-cotransporters (KCC2) (Hartmann et al., 2014; Figure 2C). CCCs do not directly consume ATP but derive energy for Cl^–^ translocation from existing cation gradients, which are established by Na^+^, K^+^ ATPase. NKCC1 mediate Cl^–^ uptake as driven by intracellular Na^+^ and K^+^ gradients, and KCC2 extrude Cl^–^ under physiological conditions through a K^+^ gradient (Chamma et al., 2012). However, as shown by Attwell and Laughlin (2001), the energetic costs of synaptic inhibition could not be directly assessed by the number of Na^+^ and K^+^ ions shuttled across CCCs. Given the high Cl^–^ load, the authors suggested that the type and level of CCC expression could be important determinants of energy expenditure during GABAergic signaling. As shown in Figure 2D, the expression of NKCC1 resulted in modest energy savings since the cation cotransport associated with Cl^–^ influx via this transporter could actually reduce the ATP consumption required to maintain cation homeostasis, given the 3:2 ratio of Na^+^ and K^+^ transport by Na^+^, K^+^ ATPase. However, compared with the absence of CCCs, the expression of KCC2 or NKCC1 together with KCC2 led to an increase in energy consumption. Thus, the authors concluded that GABAergic signaling is an important component of indirect energy expenditure through Na^+^, K^+^ ATPase function (Gao et al., 2025).

Cation-coupled chloride cotransporters are highly regulated proteins. For example, it has been demonstrated that membrane trafficking and functional expression of KCC2 is tightly controlled by the sonic hedgehog pathway, neuronal-restricted silencing element or the neurotrophin BDNF (Rivera et al., 2002). In addition, the activity of KCC2 can be modulated by phosphorylation (Kahle et al., 2013) and thereby neurotransmitters can directly interfere with the [Cl^–^]_*i*_ homeostasis (Abruzzo et al., 2021). Thus the [Cl^–^]_*i*_ is clearly regulated beyond the expression ratio of these transporters.

### 4.3 Development

In accordance with a widely described scenario in the literature, a significant transient shift in GABAergic signaling during development is associated with the expression levels of CCCs (Ji et al., 2024). This shift is characterized by a downregulation of NKCC1 and an upregulation of KCC2 (Figure 2C; Hartmann and Nothwang, 2015; Schulte et al., 2018). Progress in understanding the contribution of specific CCCs to GABAergic signaling has been made possible by using blockers of these molecules. KCC2 net transport is inhibited by furosemide (1–2 mM) (Kakazu et al., 2000; McMoneagle et al., 2024) or the selective blocker VU0463271. Other diuretic bumetanides selectively block the depolarizing GABAergic response in immature pyramidal neurons and are currently considered specific NKCC1 blockers (Xu et al., 1994; Chamma et al., 2012). In fact, in immature cells, the high [Cl^–^]_*i*_ as a function of NKCC1 activity is more negative than the Cl^–^ equilibrium (*E*_*Cl*_^–^). This characteristic makes E_*GABA*_ more depolarized than E_*M*_ (Doyon et al., 2016; Wang et al., 2002; Figure 2C). In contrast, in mature neurons, KCC2 pumps Cl^–^ out of the cells, maintaining low [Cl^–^]_*i*_. Here, the interaction of GABA with synaptic or extrasynaptic GABA_*A*_Rs leads to Cl^–^ influx into neurons and hyperpolarization of *E*_*M*_ (Ghit et al., 2021; Sallard et al., 2021).

During development the CCCs are differentially expressed in the nervous system (Hübner and Holthoff, 2013; Kirmse et al., 2018). The expression levels of NKCC1, in the contrast to KCC2, vary significantly between neurons types and stages of their development (Watanabe and Fukuda, 2015; Talifu et al., 2022). There are also evidences that the expression ratio between NKCC1 and KCC2 can be different in distinct compartments of the neurons (Virtanen et al., 2020). The most striking example is the axon initial segment, in which inhibitory synapses mediate depolarizing responses (Khirug et al., 2008), which has been linked to the delayed shift in the expression ration of Cl^–^ transporter expression in this compartment (Cherubini et al., 2022). The observation of a somatodencritic [Cl^–^]_*i*_ gradient (Kilb, 2021) suggest a variable NKCC1/KCC2 expression ratio within dendritic membranes. Thus, although an increasing KCC2 expression is correlated to Cl^–^ level changes in neurons during development, the [Cl^–^]_*i*_ is clearly regulated beyond the expression ratio of CCCs.

### 4.4 Time

The millisecond time scale ensures that synaptic neurotransmission imposes constraints on certain molecular processes and the systems that support them (Marom and Marder, 2023). After the interaction of the mediator with the receptor, the channel pore opens, and Cl^–^ enters the neurons along the gradient. This event takes several ms, after which the receptor enters a state of desensitization. Recovery of receptor conductance after desensitization can be accomplished in various ways. First, the mediator is removed from the synaptic space by GABA transporters (GAT1 and GAT3), thus swiftly terminating neurotransmission (Klepper et al., 2020). Second, the recovery of the Cl^–^ (or HCO_3_^–^) levels in the neurons takes ∼15–30 s (Karlsson et al., 2011). However, the kinetic properties of KCC2 and NKCC1 demonstrated that their time constant for the recovery of [Cl^–^]_*i*_ after GABA_*A*_R activity was more than 5 min (Kakazu et al., 2000; DeFazio et al., 2000). Thus, although it is believed that CCCs determine the ambivalent behavior of GABA_*A*_R activity by maintaining the neuronal Cl^–^ homeostasis, CCC expression is required for delayed and long-term processes to maintain neuronal [Cl^–^]_*i*_ homeostasis; however, they are not the main systems involved in the short-term recovery of anion homeostasis. These data suggest the existence of alternative mechanisms for the rapid maintenance of ionic homeostasis after receptor activity (Lombardi et al., 2019).

## 5 Role of ATP in GABA_*A*_R function

### 5.1 Run-down effect

**FIGURE 3:**
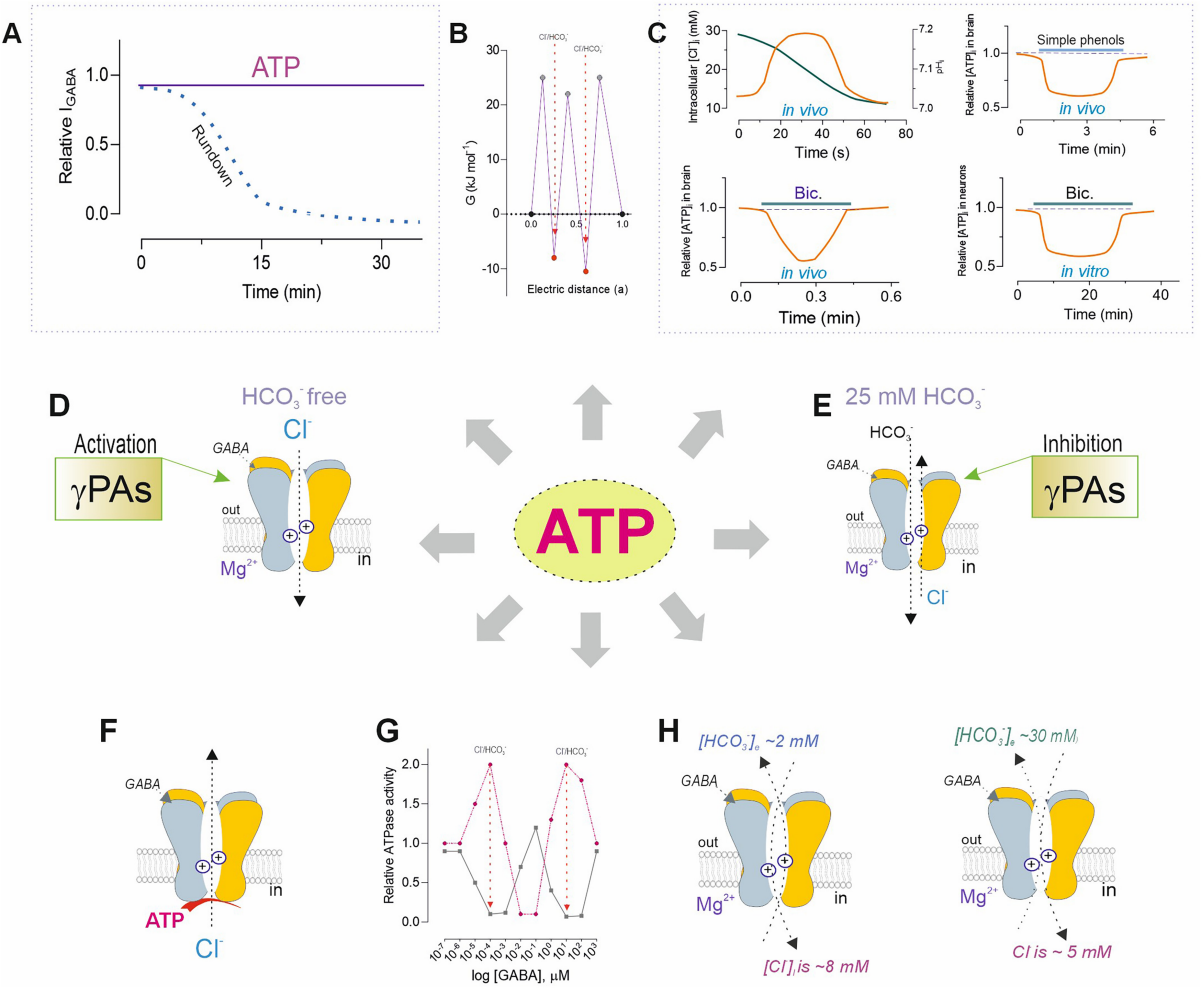
Schematic representation of ATP participation in inhibitory signaling. **(A)** ATP supports GABA_*A*_R function during the run-down effect (Stelzer et al., 1988). **(B)** Schematic representation of the charge properties of the GABA_*A*_R ionic channel. This profile was assumed to be a description of Cl^–^/HCO_3_^–^ transport across the receptor pore. Dependency of the change in free energy (kJ mol^–^) on the electric membrane distance (Bormann et al., 1987). **(C)** Drugs action on the [Cl^–^]_i_, pH_i_ and [ATP]_i_ in rodents: Upper left graph: Changes of [Cl^–^]_i_ and pH_i_ during seizure activity (Sato et al., 2017). Upper right graph: relative changes in brain ATP levels during phenol-induced convulsive activity (Angel et al., 1969). Bottom graphs: relative changes in ATP levels in the brain (left) or neurons (right) during bicuculline application (Lerchundi et al., 2020). **(D,E)** Distinct functional states of GABA_*A*_Rs after interaction with GABA in the absence or presence of HCO_3_^–^ (25 mM) in the experimental medium. Activation (Moss et al., 1995) or inhibition (Menzikov et al., 2022) of receptor activity by γPAs, respectively. **(F)** GABA_*A*_R subtypes that transport Cl^–^ against the electrochemical gradient through the phosphorylation process (Rapallino et al., 1999). **(G)** Changes in basal and Cl^–^, HCO_3_^–^ATPase activities under the influence of different concentrations of GABA. **(H)** The theoretical models of slow post-tetanic GABA_*A*_R depolarization. (Left graph) - [Cl^–^]_i_ is increased from 8 mM, whereas [HCO_3_^–^]_i_ is increased from 2 mM (Staley et al., 1995) and (right graph) - For the neurons with a high [Cl^–^]_i_, the Cl^–^ in the neurons would be depleted during the early part of the response (Perkins and Wong, 1996).

Early studies on GABA_*A*_R functional activity in neurons of various origins have demonstrated that GABA-mediated Cl^–^ conductance decreases progressively with time (Table 1). Inactivation of the GABA_*A*_Rs is eliminated by adding ATP (in the presence of Mg^2+^), indicating that this energy molecule is necessary for preventing the decline in their function (Figure 3A; Stelzer et al., 1988; Harata et al., 1997). GABA_*A*_Rs function was suggested to be maintained not only through long-term phosphorylation by PKs (e.g., PKA, PKC, CaMKII) where phosphate group are added to specific Ser/Thr or Tyr residues (phosphosites) but also by short-term phosphorylation via ATPase function (Stelzer et al., 1988; Shirasaki et al., 1992). Such fast phosphorylation of ATPase compartment refers to rapid, reversible post-translational modifications that alter an enzyme’s activity in seconds to minute. Several electrophysiological studies have shown that there are receptor subtypes that transport Cl^–^ against the electrochemical gradient via phosphorylation (Rapallino et al., 1999; Cupello and Robello, 2000). In addition, the reconstituted GABA_*A*_Rs can participate in ATP-dependent Cl^–^ transport through proteoliposome membranes (Figure 3F; Menzikov et al., 2021b, 2023).

### 5.2 γ-phosphate analogues

In their early work, Stelzer and coauthors reported that the function of GABA_*A*_R is modulated by γ-phosphate-modified ATP analogs (Stelzer et al., 1988). These ligands can inhibit the principal stages of ATP hydrolysis. For example, the nonhydrolyzable ATP analog adenylyl-imido phosphate (AMP-PNP) is used for the prehydrolysis state, and vanadate (VO_4_^3–^) and metal fluoride (MeF_*x*_) are used for the transition state (Jin Y. et al., 2017; Chase et al., 2018; Lacabanne et al., 2020). Electrophysiological and fluorescence studies have demonstrated that open and desensitized receptor states have differential sensitivities to γPAs (Menzikov et al., 2022). Specifically, vanadate (or okadaic acid) at a high concentration (100 μM) and AMP-PNP at a concentration (1 mM), activated GABA_*A*_R-mediated Cl^–^ influx into neurons by inhibiting PPs (Figure 3D; Moss et al., 1995). However, in the presence of physiological concentrations of HCO_3_^–^ (25 mM) in the experimental medium, these blockers inhibited GABA_*A*_R activity (Figure 3E; Menzikov et al., 2023).

Literary data show that the prevention of the decline in GABA_*A*_R function by ATP is primarily linked to PKs activity, whereas the reduction in receptor activity is associated with their dephosphorylation, which is mediated by PP function (Minier et al., 2000). Several studies have demonstrated the critical role of protein tyrosine kinases (PTKs) and protein tyrosine phosphatases (PTPs) (Moss et al., 1995) in the regulation of pLGICs (including, GABA_*A*_Rs). As demonstrated by several reports, genistein (a PTK inhibitor) has been shown to depress GABA-mediated Cl^–^ inflow in neuronal and recombinant GABA_*A*_Rs (Huang et al., 1999). Conversely, vanadate (a PTP and P-type ATPase inhibitor) has been observed to produce a modest increase in GABA_*A*_R activity (Clausen et al., 2016). However, PPs are inhibited by high concentrations (≥100 μM) of vanadate (Irving and Stoker, 2017), whereas P-type ATPases (for example, Na^+^, K^+^ ATPase) are highly sensitive (≤30 μM) to vanadate (Searle et al., 1983). While in the presence of physiological concentrations of HCO_3_^–^ in the experimental medium, vanadate inhibited GABA_*A*_-evoked Cl^–^ inflows in the low concentration range (≤30 μM), indicating the involvement of ATPase activity in this process.

### 5.3 Massive activation

**FIGURE 4:**
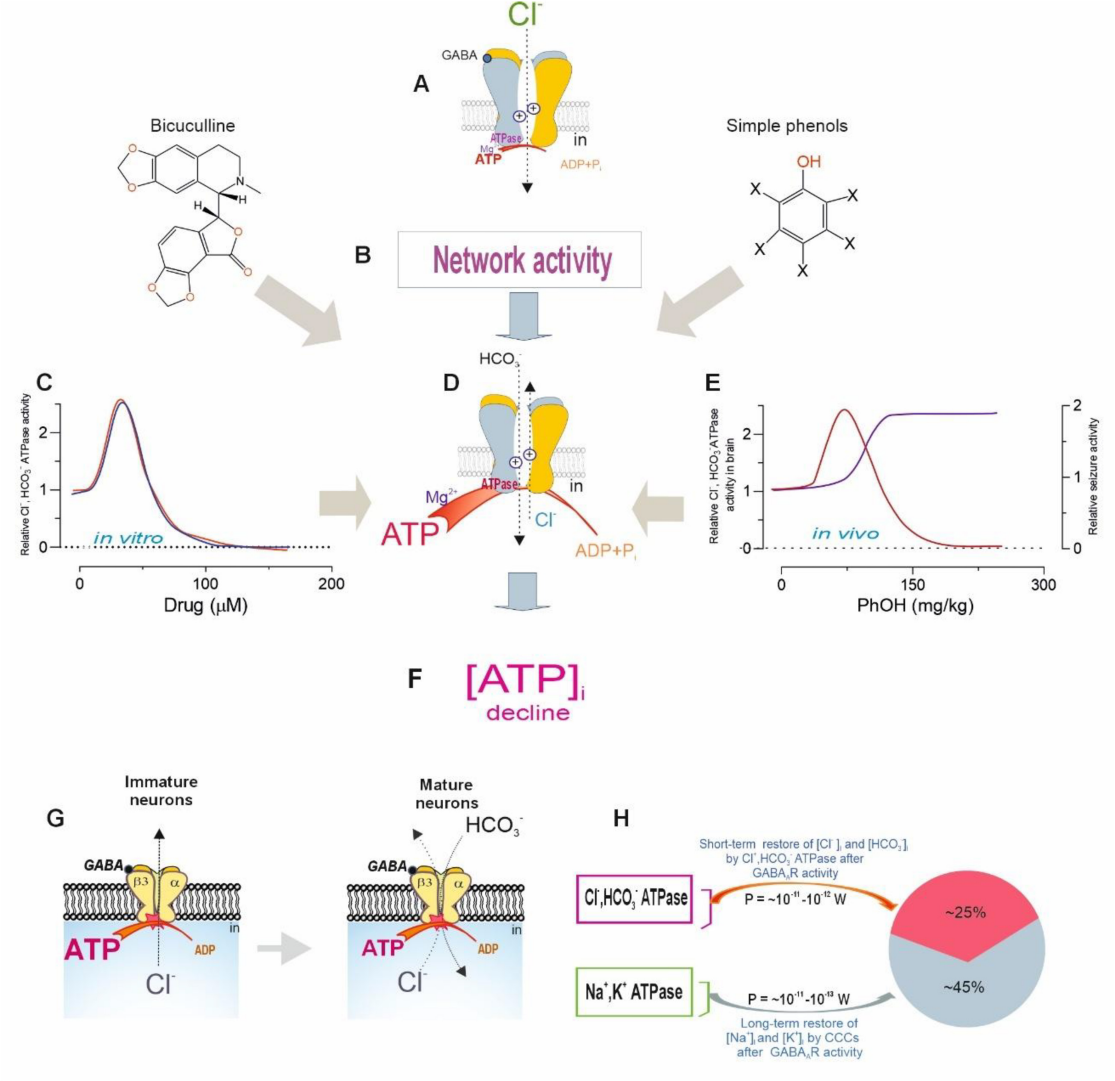
Hypothetical scheme for the direct involvement of GABA_A_ receptors in energy consumption during network activity. **(A)** GABA_*A*_R_ATP_ subtypes in a normal (or basal) state have low ATPase activity and, when interacting with a mediator, induce Cl^–^ entry into neurons and hyperpolarization of *V*_*m*_. **(B)** GABA_A_ receptors have binding sites for bicuculline and simple phenols (Bormann et al., 1987). **(C)** During drug-mediated network activity, GABA_*A*_R_ATP_ subtypes mediate the increase in [Cl^–^]_i_, decrease in pH_i_ and increase in ATPase activity. As a result, there is a depolarization of *V*_*m*_. **(D)** *In vitro* experiments showed that bicuculline or phenol (PhOH) exerts a sinusoidal effect on the ATPase activity; at low concentrations they activate the enzyme, whereas at high concentrations, they inhibit it. **(E)** The administration of phenol *in vivo* experiments also showed that at low concentrations activated the ATPase activity in the rodents brain, whereas at high concentrations, it completely inhibited the activity. **(F)** During network activity, a decrease in ATP level in the neurons by ATPase function, followed by a decrease in receptor activity. **(G)** Differential role of ATPase in undeveloped and developed neurons. In immature neurons, GABA_*A*_Rs are forced to use ATP energy for Cl^–^ transport against an electrochemical gradient. In contrast, GABA_*A*_Rs in the adult brain show a distinct pattern. They will only use the energy of ATP in the presence of physiological concentrations of bicarbonate efflux of HCO_3_^–^ from neurons and increase the Cl^–^ level in cells. **(H)** Two systems are involved in ATP consumption in different ways: indirectly the Na^+^, K^+^ ATPase and directly the Cl^–^, HCO_3_^–^ATPase.

It is generally accepted that the short activation of GABA_*A*_Rs by mediator usually leads to rapid Cl^–^ influx into neurons and, as a result, to hyperpolarization of *V*_*m*_ (Figure 4A). However, more than three decades ago it was shown that during their massive activation there was a depolarization of *V*_*m*_ (Figure 2A; Branchereau et al., 2016). The dual nature of the GABA response involves a fast (*ms*), initial hyperpolarizing component followed by a depolarizing phase that can last several seconds (Perkins and Wong, 1996; Staley and Proctor, 1999). The major mechanism behind this depolarization is attributed to the accumulation of intracellular Cl^–^ and depolarize the Nernst potential. When the *V*_*m*_ is relatively hyperpolarized, this will result in a Cl^–^ efflux instead of influx trying to reach their depolarized equilibrium potential. Detailed analysis of the ionic basis of the biphasic responses showed that the initial part of the depolarization was caused by anionic redistribution, in which the inward HCO_3_^–^ current drove a depolarization that promoted an increase in [Cl^–^]_*i*_ and, as a result, led to a positive shift in *E*_*GABA*_. Although Cl^–^ is the major permeant anion via ion channel pores, inhibitory receptors are also permeable for HCO_3_^–^, with a permeability ratio of Cl^–^ to HCO_3_^–^ of between ∼0.2 and 0.5 (bicarbonate flows in the opposite direction) (Perkins and Wong, 1996). In contrast to Cl^–^, HCO_3_^–^ invariably mediates only a depolarizing current that is attenuate because of dissipating HCO_3_^–^ and pH gradients (Fatima-Shad and Barry, 1993). Thus, the GABA_*A*_-mediated regulation of the intracellular bicarbonate concentration ([HCO_3_^–^]_*i*_) and, as a result, the shift in the intracellular (pH_*i*_) are essential factors that also determine the molecular properties of receptor- and GABA_*A*_-mediated Cl^–^ conductance (Lombardi et al., 2019; Sato et al., 2017).

Several theoretical models have been proposed to explain the ionic basis of slow post-tetanic GABA_*A*_R depolarization. Specifically, GABA_*A*_-induced depolarization potential is dependent on the preservation of HCO_3_^–^ and the collapse of Cl^–^ gradients (Staley and Proctor, 1999). The authors suggested that during depolarization, the [Cl^–^]_*i*_ increased from 8 mM, whereas the [HCO_3_^–^]_*i*_ increased from 2 mM (Figure 3H, left graph) (Staley et al., 1995). Another model contradicts the Cl^–^ accumulation hypothesis and suggests that “distinct” GABA_*A*_R subtypes with special permeability for anions, namely, lower Cl^–^ and higher HCO_3_^–^ permeability through the receptor pore and slower kinetics, are present (Perkins and Wong, 1996). The authors suggest that for the neurons with a high [Cl^–^]_*i*_, the Cl^–^ in the neurons would be depleted during the early (inward current) part of the response, which would have to occur at a comparable rate with Cl^–^ accumulation, and the polarity of the late response would be determined by the HCO_3_^–^ gradient (Figure 3H, right graph). In this hypothetical model, the role of a putative Cl^–^ATPase in the restoration of [Cl^–^]_*i*_ after receptor activity was considered. However, the authors were unable to establish what types of receptors were present (Perkins and Wong, 1996; Perkins, 1999). Recent molecular studies support the existence of GABA_*A*_R β3 subtypes with high HCO_3_^–^ permeability that could be responsible for depolarizing GABA_*A*_-mediated responses (Menzikov et al., 2023).

Because the [Cl^–^]_*i*_ cannot be reliably measured depending on the expression of CCCs and the studies of the Cl^–^ concentration is not accurate since the *in vitro* research do not take into consideration the network activity, the quantum model was proposed by some authors (Nawafleh et al., 2022). The quantum tunneling model of ions is applied on GABA receptors and their corresponding chloride ions to show how Cl^–^ can depolarize the resting membrane potential. The quantum model states that intracellular Cl^–^ have higher quantum tunneling probability than extracellular Cl^–^. This is attributed to the discrepancy in the kinetic energy between them. At physiological parameters, the quantum tunneling is negligible to the degree that Cl^–^ cannot depolarize the membrane potential. Under certain conditions such as early neuronal development, gain-of-function mutations, stroke and trauma that can lower the energy barrier of the closed gate of GABA receptors, the quantum tunneling is enhanced so that the Cl^–^ can depolarize the resting membrane potential.

Understanding these processes is complicated by the fact that HCO_3_^–^ can modulate neuronal activity in a potentially pH-independent manner by activation of cyclic nucleotide signaling or PKs (Chen et al., 2000; Steegborn et al., 2005), by influencing membrane potential by flux through Cl^–^ channels or transporters (Hamidi and Avoli, 2015; Gonçalves and Mulkey, 2018). In addition, [Cl^–^]_*i*_ is also directly influenced by GABAergic activity via Cl^–^ fluxes (Raimondo et al., 2012; Branchereau et al., 2016). Specifically, GABA_*A*_-mediated depolarizing responses can be observed under pathophysiological conditions like trauma, epilepsy or stroke (Kilb, 2021). Specifically, in hyperexcitable states (epilepsy), intense neuronal firing can overload neurons with Cl^–^, leading to prolonged GABA-mediated depolarizations that can trigger seizures. This is not only relevant for high, pathophysiological activity patterns, but also for physiological levels of neuronal activity (Currin et al., 2020). *In silico* experiments show that these activity-dependent [Cl^–^]_*i*_ changes are influenced by the dendritic morphology, membrane properties as well as the kinetics of GABAergic inputs, anion homeostasis (Doyon et al., 2016; Lombardi et al., 2019). In addition, such activity-dependent [Cl^–^]_*i*_ transients are augmented by coincident glutamatergic inputs (Lombardi et al., 2021). The impact of neuronal activity on [Cl^–^]_*i*_ is particularly relevant under *in vivo* situations, where an ongoing “bombardment” with GABAergic and glutamatergic synaptic inputs has been suggested (Steriade et al., 2001).

On the other side, the transmembrane transporters implicated in bicarbonate/pH (Ruffin et al., 2014) and chloride regulation include Na^+^/H^+^ exchangers (NHE), Na^+^-independent/dependent Cl^–^/HCO_3_^–^ exchangers, and Na^+^/HCO_3_^–^ cotransporters (Figure 2B; Casey et al., 2009; Liu et al., 2015; Pressey et al., 2023; Kourosh-Arami and Komaki, 2026). Literature shows that stilbene derivatives [for example, 4-acetamido-4′-isothiocyano-2,2′-disulfonic stilbene (SITS) or 4,4′-diisothiocyano-2,2′-disulfonic stilbene (DIDS)] inhibit Cl^–^/HCO_3_^–^ exchange transport performed by various membrane structures when they are present at concentrations of 100–300 μM (Svichar et al., 2011; Salameh et al., 2017). These studies suggest that ATPase, which may be involved in the restoration of anion levels after receptor activity, must be activated by Cl^–^ and HCO_3_^–^ and be sensitive to vanadate and stilbene derivatives (Wu et al., 2025).

### 5.4 Glucose deficiency syndrome

Impairments in glycolysis directly compromise GABA_*A*_Rs function, as receptor phosphorylation and stability depend on ATP produced by membrane-associated glycolysis enzymes (Laschet et al., 2007; Hartman, 2017). Moreover, in epilepsy, impaired glycolysis (specifically reduced ATP production) leads to the dysfunction of GABA_*A*_R, reducing inhibitory tone and increasing excitability (Hartman, 2017). Moreover, GLUT1-DS primarily affects inhibitory signaling (Klepper et al., 2020; Fischer et al., 2022). Specifically, in a mouse model of GLUT1-DS, the authors showed no energy metabolism impairments, whereas the synaptic inhibition of cortical pyramidal and thalamic cells was reduced, confirming the important role of energy substrates in maintaining their functional activity (Rajasekaran et al., 2022).

## 6 GABA_*A*_R_*ATP*_ as candidate for direct ATP consumption

### 6.1 Enzyme features

The molecular studies have directly shown that β subunits (specifically, β3 and β1 subunits), in the contrast to α and γ subunits are catalytic, and determines the ATP hydrolysis (GABA_*A*_R_*ATP*_). The ATPase activity can be optimally detected either in the presence of low anion concentrations (∼10 mM Cl^–^ and ∼2 mM HCO_3_^–^) or in the range of their high concentrations (∼5 mM Cl^–^ and ∼25 mM HCO_3_^–^) (Menzikov et al., 2021a, 2023). These anion concentrations are similar to those described in hypothetical models of their transport through the receptor pore during GABA_*A*_R-mediated depolarization (Staley et al., 1995). The stimulating the enzyme activity anions are Cl^–^ > Br^–^ > I^–^ > F^–^, and also HCO_3_^–^, SO_3_^2^, while NO^2–^ inhibits the enzyme and no effect elicited by NO_3_^2–^. The Na^+^, K^+^ and choline cations have no effect on the enzyme. Vanadate inhibits the Cl^–^, HCO_3_^–^ATPase, similar as other transport ATPases, in the range of low concentrations (10–25 μM). In addition, the Cl^–^, HCO_3_^–^ATPase is inhibited by AMP-PNP (≥1 mM) or AlF_2_ (20 μM) (Menzikov et al., 2021b). Blockers of ionic channels: Furosemide inhibits the enzyme in the range of concentration at 0.5–1 mM. SITS or DIDS are inhibitors of anion exchangers (Cl^–^\HCO_3_^–^) (like Band 3) in the range concentration of 0.2–0.5 mM. Picrotoxin (100 μM) and bicuculline (25 μM) inhibit the Cl^–^, HCO_3_^–^ATPase activity as well as the effect of GABA on the enzyme.

The GABA_*A*_R_*ATP*_ subtypes differentially activated by anions during neuronal development. Specifically, during the first week of post-embryonic development of the rat, cortical ATPase showed a dominant role for Cl^–^ activation, whereas after postnatal day 10, a dominant role for HCO_3_^–^ and a minor role for Cl^–^ became apparent in the activation of the enzyme (Menzikov et al., 2023). Moreover, it was established that Cl^–^ ATPase is mostly present in the β1 subunit, while Cl^–^, HCO_3_^–^ATPase belongs to the β3 subunit. These data are similar to those reported in the literature, both in terms of the distinct properties of the subunits and the changes in their trafficking to the cell surface (Goetz et al., 2007). In particular, a significant expression of GABA_*A*_R β1 subunit mRNA in neurons isolated from samples taken between days P5 and P7 as opposed to the β2 or β3 subunits (Brooks-Kayal et al., 2001) was found, and neurons isolated from samples taken from postnatal days 17–21 showed an increase in the expression of GABA_*A*_R β2 and β3 subunit mRNA. In addition, in immature neurons with high [Cl^–^]_*i*_, HCO_3_^–^ produced a minimal effect on *E*_*GABA*_ (Raimondo et al., 2012).

The ATP in the presence of Mg^2+^ is the most effective hydrolyzed nucleotide (ATP > UTP > CTP > ADP). Other divalent cations can replace Mg^2+^ in the following order: Mg^2+^ > Mn^2+^ > Co^2+^ > Al^2+^ > Cd^2+^. Addition of Mg^2+^ATP in an experimental medium, the ATPase phosphorylates the receptor and maintains its function. The β3 (or β1)-containing GABA_*A*_R ensembles can catalyze the auto-phosphorylation of a high-energy aspartate residue during ATP hydrolysis (Menzikov et al., 2021b). It was proposed the following model for ATPase function − (i) the first stage of ATP hydrolysis in the presence of Mg^2+^ is its joining with enzyme; (ii) formation of macroergic phosphorylated intermediate, inhibitors are hydroxylamine, o-vanadate or oligomycin; (iii) the third stage is dephosphorylation by anions (Cl^–^ and HCO_3_^–^) and GABAergic ligands. The Cl^–^, HCO_3_^–^ATPase activity in the plasma membranes fraction from the brain of rodents is ∼0.01 μmol P_*i*_⋅s^–1^⋅mg^–1^, and the purified enzyme has activity ∼35.0 μmol P_*i*_⋅s^–1^⋅mg^–1^.

### 6.2 Sinusoidal nature of enzymatic behavior and receptor energy profile

The activity of GABA_*A*_R_*ATP*_ subtypes consists of basal activity and the activating effect of anions. GABAergic ligands have an allosteric effect on the both enzyme activity and the receptor function (Sieghart, 2015). Specifically, in the presence of GABA in the experience medium in a wide range of concentrations, with an increase in the basal activity, the Cl^–^, HCO_3_^–^ATPase activity decreases, and vice versa. As shown in Figures 3B, G, the sinusoidal character of the ATPase activity in the absence or presence of Cl^–^ and various GABA concentrations is similar to the schematic representation of Cl^–^ transport via the receptor pore (Harata et al., 1997). Specifically, the GABA_*A*_Rs function in rat hippocampus shows the dual nature of the GABA effect on the membrane potential, depending on its concentration. At low (0.01 μM) and high (1-10 μM) concentrations the neurotransmitter causes hyperpolarization of neurons, while at average range concentrations (0.1-10 μM) it is not effective (Plinkert et al., 1993; Harata et al., 1997). The energy profile of the receptor ion channel is shown in Figure 3B, the potential failures are also consistent with the binding site of Cl^–^ and/or HCO_3_^–^ ions with the ion-channel (Bormann et al., 1987). The decrease in the basal activity is associated with the appearance of enzyme activation by anions during different pre-incubation times of GABA_*A*_R_*ATP*_ with ligands. In addition, GABAergic ligands (as example, propofol or neurosteroids) have also recently been shown to modulate the energetic state of inhibitory receptors (Borghese and Goldschen-Ohm, 2024; Pierce et al., 2024).

Thus, GABA_*A*_R_*ATP*_ subtypes can exist either in a phosphorylated or dephosphorylated form. The former case has a low basal Mg^2+^ATPase activity and its activated by Cl^–^ and HCO_3_^–^ ions. This state enables the protein to participate in the ATP-dependent transport of anions. Phosphorylation is opposed by a dephosphorylation process which is catalyzed by a vanadate-sensitive phosphatase. In this case, it has a high basal Mg^2+^ATPase activity and it is not activated by anions. However, the question arises here as to How exactly does ATP hydrolysis couple to ion movement in a channel structurally shaped like a passive pore (Miller et al., 2017; Dyla et al., 2017)? Currently, structures are known that possess both the properties of an ion channel and intrinsic ATPase activity; for example, the cystic fibrosis ion channel (CFTR) (Hwang and Kirk, 2013). CFTR belongs to the ATP-binding cassette (ABC) transporter family, and functions as an ion channel rather than an active pump (Horvat et al., 2006). While other ABC transporters function as transport ATPases. Unlike ligand-gated channels, CFTR consumes ATP to open and close the channel pore and drive the conformational changes that regulate the passage of chloride and bicarbonate ions.

On the other hand, receptor structures demonstrate the ability to form an acid-stable phosphorylated intermediate that is sensitive to hydroxylamine. Indeed, hydroxylamines as potent nucleophiles attack and cleave the specific phosphate-nitrogen or phosphate-carbon bonds. The high-energy acyl-phosphate bond (mixed anhydride) is formed by linking a carboxylic acid to a phosphate group (Chang et al., 2018). Such unique chemical profile is the hallmark of transient, energy-transferring intermediates (aspartyl-phosphate) in enzymes such as P-type ATPases (Viola et al., 1989; Horvat et al., 2006). It is characterized by a large, negative standard free energy of hydrolysis (releasing ∼49 kJ/mol) and plays a vital role in biological energy storage and transfer (Saile and Pappas, 2026). Since GABA_*A*_Rs are ligand-gated ion channels (ionotropic receptors), it might be speculated that switching from prolonged to brief receptor phosphorylation at allosteric action of agonist is required for selective ion permeability. Moreover, such transitive switches may be required to modulate and facilitate inhibitory signaling during network activity or control structural changes.

## 7 Network activity and “ATPase” hypothesis

GABAergic neurotransmission is fundamental to the synchronization of neuronal activity (Buchin et al., 2018; Khazipov, 2016). Reduced energy balance in nerve cells is widely acknowledged to be associated with neurological disorders (such as epilepsy, tremors, etc.) (Clemente-Suárez et al., 2023). Neuronal ATP levels also decrease during the induction of network/seizure activity (Du et al., 2008; Rangaraju et al., 2014; Lerchundi et al., 2020; Figure 4B). Specifically, energy production (P_*i*_/min/g) increased significantly with seizure-simulating activity and decreased with phenobarbital anesthesia (Winn et al., 1980; Walton et al., 1998). The argument that the cost of removing Cl^–^ from neurons via CCCs benefits neural coding does not account for the increase in Cl^–^. However, the occurrence of this event during network/seizure activity would require rapid restoration of chloride homeostasis through direct energy expenditure by ATPase. At the same time, other studies indicate the involvement of adenosine receptors in epileptic activity through interaction with GABA_*A*_R (Roseti et al., 2008).

### 7.1 Network/seizure activity, [Cl^–^]_*i*_ and pH_*i*_ changes

Massive receptor activation during epileptiform discharge (ED) causes depolarizing shifts in the *E*_*GABA*_ of hippocampal pyramidal neurons (Mazzuferi et al., 2010; Fujiwara-Tsukamoto et al., 2003; Svichar et al., 2011), which can alter the activity of GABA_*A*_Rs such that they begin to promote rather than oppose epileptiform activity (Kim and Yoon, 2017; Khazipov, 2016). Specifically, during epileptic seizures, the stable depolarizing drive of GABA_*A*_R-mediated HCO_3_^–^ currents increases activity-dependent Cl^–^ uptake and thus directly contributes to the generation of excitation under these conditions (Isomura et al., 2003; Fei et al., 2020). Fluorescence and optical genetic methods have also demonstrated essential increases in [Cl^–^]_*i*_ and pH_*i*_ during neurological disorders (Alberio et al., 2025; Sato et al., 2017; Cãlin et al., 2023) and increased network activity (Burman et al., 2024; Figure 3C, upper left graph).

### 7.2 Bicuculline induces [ATP]_*i*_ decline

Various approaches to monitoring cellular ATP levels have suggested that neurons experience a decrease in neuronal ATP levels following the application of glutamate or the induction of network activity (Gerkau et al., 2019; Vergara et al., 2019). Of particular interest are the literature data that demonstrate energy metabolites and their changes during various manipulations of neurons during network activity (Walton et al., 1998). Specifically, *in vivo* studies revealed a reduction in ATP levels in the brains of rodents during the first 60 s of bicuculline-induced seizure activity (Figure 3C, bottom left graph) (Winn et al., 1980). Other reports have also shown that the inhibition of GABA_*A*_Rs by the application of bicuculline (10 μM) and the activation of NMDA receptors by reducing the extracellular Mg^2+^ concentration to 0.5 mM induce recurrent neuronal network activity in hippocampal brain slices (Figure 3C, bottom right graph) (Mele et al., 2021; Buchin et al., 2018). Recent studies in neuronal slices expressing the ATP sensor (ATeam1.03YEMK) have shown that inhibition of GABA_*A*_Rs by the application of bicuculline (10 μM) and reduced Mg^2+^ induces recurrent neuronal network activity (Lerchundi et al., 2020). While [ATP]_*i*_ in neurons rapidly decreased by an average of ∼0.3 mM, as determined after 2 min of exposure (Gerkau et al., 2019), [ATP]_*i*_ continued to decrease, reaching ∼0.7 mM below baseline at 5 min and ∼1 mM at 10 min. The underlying reason for the decrease in [ATP]_*i*_ during this type of neuronal activity is still largely unknown. *In vivo* studies on the properties of GABA_*A*_R_*ATP*_ have shown that bicuculline increases GABA_*A*_R activity and ATPase activity at low concentrations (1–10 μM) but completely inhibits them at high concentrations (Ueno et al., 1997; Figures 4C,D).

### 7.3 Simple phenols induce [ATP]_*i*_ fall

The chemicals with a dialkylated phenyl ring are positive allosteric modulators of the GABA_*A*_Rs (Menzikov et al., 2024); however, only chemicals with a 1-hydroxyl group attached to the benzene ring can directly activate receptors. For example, phenol (10 mM) activated oocyte-expressed GABA_*A*_R-induced Cl^–^ currents (Brosnan and Pham, 2018). Additionally, of the monosubstituted benzene compounds, only the simple phenols (SPs) were active in producing myoclonic convulsions in mice, suggesting that the phenolic hydroxyl group is a basic requirement for this process (Angel and Rogers, 1972). Specifically, administration of SPs such as catechol and pyrogallol at 60 mg/kg produced convulsive activity together with decreased brain ATP concentrations in mice (Angel et al., 1969). The maximum effect of SPs occurs 3–4 min after injection and then rapidly decreases (Figure 3C, upper right graph). The reduction in neuronal ATP levels during the seizure activity was slightly related to mitochondrial enzymes changes; however, the main cause of this change remains largely mysterious (Walton et al., 1998). Recent *in vivo* and *in vitro* studies have shown that the Cl^–^, HCO_3_^–^ATPase, is modulated by SPs in a concentration-dependent manner (activated or inhibited) and is involved in seizure activity (Figure 4E).

### 7.4 Hypothesis of direct energy expenditure by GABA_*A*_R_*ATP*_ subtypes

An analysis of the literature data revealed that the receptor subtypes containing β3 (or β1) subunits exhibit ATPase properties. In this context, among the significant facts confirming the significant role of GABA_*A*_R function in energy expenditure is (i) a decrease in receptor function in the absence of ATP or in the context of GLUT1-DS; (ii) inhibition of GABA_*A*_-mediated Cl^–^ inflow into neurons in the presence of bicarbonate by γPAs; and (iii) the presence of receptor subtypes that are involved in Cl^–^ transport against the electrochemical gradient through phosphorylation processes; (iv) the presence of an ATPase compartment in the receptor structure; and (v) an increase in ATPase activity during network activity and a decrease in [ATP]_*i*_ (Figures 3, 4F).

The presence of the ATPase in the structure of β1/β3-containing receptors requires additional adjustments to assess the role of GABA_*A*_Rs in the expenditure of ATP energy. In immature neurons, GABA_*A*_Rs are forced to use ATP energy for Cl^–^ transport against an electrochemical gradient (Figure 4G, left graph). After the mediator binds with receptor and Cl^–^ is released from the neurons, GABA_*A*_R_*ATP*_ consumes ATP again to restore neuronal chloride homeostasis. In contrast to immature neurons, GABA_*A*_Rs in the adult brain show a distinct pattern. They will only use the energy of ATP in the presence of physiological concentrations of bicarbonate (Figure 4G, right graph).

Thus, during GABA_*A*_R signaling, two systems are involved in ATP consumption in different ways: indirectly–Na^+^, K^+^ ATPase and directly–GABA_*A*_R_*ATP*_ (Cl^–^, HCO_3_^–^ATPase) (Figure 4H). The literature demonstrates that a single Na^+^, K^+^ ATPase consumes power on the scale of 10^–11^–10^–13^ W, dissipating heat and driving ion flow, whereas cell pump consumes energy at an extremely low rate in the range of 10^–14^–10^–17^ W depending on metabolic activity, dissipating power (*P* = R_ATP_ × ΔG, where R_ATP_ is the ATP consumption rate mol/s and ΔG is the Gibbs free energy of ATP in J/mol) (Bo et al., 2023). In turn, the Cl^–^, HCO_3_^–^ATPase activity in the virus-like particles (VLPs) of the recombinant (α2β3γ2) GABA_*A*_Rs is ∼0.001 μmol^–1^ P_i_⋅s^–1^ and the purified receptor had the ATPase activity of about 0.1 μmol^–1^ P_i_⋅s^–1^ (Menzikov et al., 2021b; Figure 4H). The calculated power can be about 10^–11^–10^–12^ W. However, due to the allosteric effect of GABAergic ligands on receptor function, the activity of the Cl^–^, HCO_3_^–^ATPase can also alter in an allosteric manner. Probably, the sinusoidal cycle likely plays a significant role in modulating receptor function through conformational and phosphorylation-mediated changes.

## 8 Limitations for ATPase involvement

It should be noted right away that the current data are correlative. Despite the existence of GABA_*A*_R subtypes that have an ATP hydrolase compartment, the assumption that they are involved in direct energy expenditure has several limitations. This could be due to several factors. First, there are limitations to the methodological approaches in the study of receptor properties. In particular, although GABA_*A*_R_ATP_ subtypes can participate in ATP-dependent Cl^–^ transport across proteoliposome membranes, there are no experimental data for direct involvement of the enzyme in Cl^–^ transport against the electrochemical gradient or a clear stoichiometry of anion transport through the receptor channel. Furthermore, links between GABA_*A*_R-ATPase activity and seizure-related ATP decline remain largely correlative, without isolating this mechanism from general metabolic stress or other ATP-consuming processes. Secondly, the role of bicarbonate in the functional activity of receptors is not fully understood. Lastly, the ATPase hypothesis contradicts the generally accepted data on the structural and functional properties of inhibitory receptors and does not have a generally accepted evidence base. Indeed, structural studies have convincingly shown that the receptor subunits form an ion channel pore for passive permeability of ions along a gradient (Miller et al., 2017). Therefore, it is difficult to conclude the role of phosphorylation during ATP hydrolysis and subsequent receptor conformational rearrangements (Menzikov and Morozov, 2019). Moreover, a lack of data demonstrating steps of structural changes during ion transport as well as specific inhibitors of the enzyme do not allow us to identify which known ATPase families such an enzyme compartment may belong to Locher (2016).

### 8.1 Counterarguments by structural biology

There are nineteen different subunits that can form pentameric receptor ensembles; however, in the vertebrate brain, the most common configuration is 2α:2β:1γ (Treviño et al., 2025). Recent cryo-electron microscopy (cryo-EM) studies have elucidated diverse assembly configurations involving the α1, α2, α3, β1, β2, β3, and γ2 subunits, including receptors featuring a β-β interface (Laverty et al., 2019; Sun et al., 2026). Each subunit contains a large hydrophilic extracellular N-terminus, four hydrophobic transmembrane domains (TMD: TM1–TM4), and a small extracellular C-terminus. TM1 and TM2 are connected by a short intracellular loop, whereas a short extracellular loop connects TM2 and TM3. In addition, TM3 and TM4 are connected by a lengthy intracellular loop that can be phosphorylated. Structural studies have convincingly shown that the receptor subunits form an ion channel pore for the passive permeability of ions along a gradient without the involvement of ATP hydrolysis (Miller and Aricescu, 2014). The cryo-EM maps are foundational for understanding the gating mechanisms of the receptor pore and the binding of various modulators (Fan et al., 2025). Moreover, these studies were carried out without the involvement of ATP in passive permeability of Cl^–^ through the ion channel pore (Figure 4A).

The β3 subunits can form homomeric pentamers, which have been found at extrasynaptic locations. Specifically, cryo-EM studies of homomeric GABA_*A*_R β3 have provided high-resolution insights into the structure and function of these receptors, revealing them as pentameric ion channels that can form in the absence of α or γ subunits. The GABA_*A*_R β3 homomer is a pentameric assembly with five subunits that forms a cylinder-shaped central ion channel (Sun et al., 2026). Each subunit contains a large N-terminal extracellular domain (ECD) and a C-terminal four-helix bundle transmembrane domain (TMD). Moreover, these studies reveal the interaction of H267 with a glutamate (E270) in the neighboring subunit M2 helix, forming a ring structure that stabilizes the transmembrane pore. The β3 homomer in the resting state shows a constricted ion pore at the level of the M2 helix, specifically at the Ala 248 position, which restricts ion conduction, representing a closed state.

### 8.2 Counterarguments by physiology

The functional characteristics of cys-loop receptors are such that they directly mediate the binding of a mediator to the opening of an ion channel pore, which is formed by subunits (Miller et al., 2017). These events facilitate the process of passive Cl^–^ transport into neurons along the ion gradient, with subsequent accumulation of negative charge and, as a result, hyperpolarization of the *E*_*M*_. The impact of GABA_*A*_Rs on cellular electrophysiology mostly depends on the time course and amplitude of the Cl^–^ currents evoked by GABA (Ghit et al., 2021; Sallard et al., 2021). Typically, the current time course following the introduction of an agonist in the receptor environment consists of an initial current peak, followed by a decrease in amplitude. This decrease ends with stabilization at lower amplitudes or complete closure of the receptors, depending on the agonist concentration, duration of exposure to the agonist, and receptor subtype. The conductance and Cl^–^ current depend on the subunit composition, the ionic conditions of the environment and the patterns of GABA exposure they face. This consideration in turn ensures precise modulation and diversity of GABA responses.

## 9 Conclusion

Although the brain is an energy-consuming organ even in a basal state, under certain conditions, the energy expenditure of the CNS can increase sharply, which imposes special restrictions on the balanced functioning of the entire organism (Natsubori et al., 2020; Pepperell, 2024). Therefore, identifying structures and processes that can increase energy budget expenditure in neurons during network activity is crucial for addressing the consequences of energy deficiency (Faria-Pereira and Morais, 2022). The literature shows that synaptic transmission is most energy expensive, whereas the energy budget of neurons is limited (Attwell and Laughlin, 2001; Lennie, 2003; Howarth et al., 2012). This in turn requires coordination between neural activity and metabolic energy levels during synaptic transmission (Khatri and Man, 2013).

During synaptic transmission, the higher neurotransmitter release probability of synapses means that more information can be transmitted to postsynaptic neurons. However, many studies have shown that most synapses maintain a relatively low release of neurotransmitter probability to obtain optimal energy efficiency (Goldman, 2004; Volgushev et al., 2004; Harris et al., 2012). Moreover, various receptor subtypes have varying sensitivities to GABA, and their subunit composition can influence their overall function. On the other side, GABA_*A*_Rs can become less responsive (desensitized) to GABA, but factors that prevent or delay this process can result in the accumulation of mediator (Burman et al., 2024). In turn, high concentrations of mediator in the extracellular space may be the main cause of massive activation and as result, the network activity. Subsequent ATP decline during network activity causes a decline in their functional activity, resulting in neuronal excitation.

In the CNS, the information is coded by changes in membrane potential–depolarization (amplification to a more positive intracellular voltage) and hyperpolarization (amplification to a more negative voltage) or an increase in cell conductance with no change in voltage (Li et al., 2023; Levy and Calvert, 2021; MacVicar et al., 2017). The regulation of neuronal excitability by inhibition is critically important for controlling the range of summed excitatory input strengths. However, it is believed that the energetic consideration, which is the reason for favoring computation with both excitation and inhibition, is that inhibition may allow information to be represented with less energy use because inhibition can alter the membrane potential without the large influx of Na^+^ ions (that must then be pumped out, consuming energy by Na^+^, K^+^ ATPase), which is needed for excitatory synapses (Cordeiro et al., 2024; Gerkau et al., 2019). However, these arguments do not take into account the rapid change in bicarbonate/proton è chloride ions levels during network activity. Such events require the presence of a structure that will adjust to these changes. A possible candidate for the role of such structure may be Cl^–^, HCO_3_^–^ATPase. Further study of the role of the ATPase in the molecular and structural mechanisms of inhibitory receptor functioning will help finally clarify its direct contribution to energy consumption.

## Data Availability

The original contributions presented in this study are included in the article/supplementary material, further inquiries can be directed to the corresponding author.
